# Rice-Grain Calcifications: A Classic Sign of Muscular Cysticercosis

**DOI:** 10.7759/cureus.116619

**Published:** 2026-09-21

**Authors:** Daniela Soares, Iara Ferreira, Joana Sequeira

**Affiliations:** 1 Internal Medicine, Unidade Local de Saúde Entre Douro e Vouga, Santa Maria da Feira, PRT

**Keywords:** cysticercosis, muscle, myalgia, radiography calcification, skeletal

## Abstract

Muscular cysticercosis is an uncommon manifestation of *Taenia solium* infection, usually discovered incidentally, that produces characteristic elongated intramuscular calcifications on plain radiographs. We describe an 85-year-old man admitted with a viral respiratory infection complicated by respiratory failure, who developed myalgia disproportionate to the infection. Plain radiographs of the lower limbs, obtained to investigate the myalgia, revealed numerous “rice-grain” calcifications parallel to the muscle fibers, consistent with the patient's known history of cysticercosis. Recognizing this pattern as representing chronic, inactive disease avoided unnecessary investigations and antiparasitic treatment. The myalgia was not attributable to the calcified lesions and was instead attributed to a combination of the patient's advanced age, hospitalization-related immobilization, and the viral infection itself, deep vein thrombosis having been excluded. Symptoms resolved within one week with paracetamol alone. This case highlights the importance of correctly attributing incidental radiographic findings so as not to delay recognition of the true cause of a patient's symptoms.

## Introduction

Taeniasis and cysticercosis are distinct manifestations of *Taenia solium* infection. Taeniasis results from ingestion of undercooked pork containing viable cysticerci and is caused by the adult tapeworm in the human intestine. By contrast, cysticercosis develops after ingestion of *T. solium* eggs through fecal-oral transmission; the released oncospheres penetrate the intestinal wall, disseminate hematogenously, and develop into larval cysts in tissues [1]. The disease remains endemic in several regions of Latin America, sub-Saharan Africa, and Asia, while most cases reported in Western Europe are associated with migration or travel to endemic areas [2,3]. Neurocysticercosis is the most common and clinically significant presentation, but the larvae may lodge in skeletal muscle, where isolated involvement is uncommon and typically asymptomatic. Tissue cysticerci progress from a viable vesicular stage through degenerative stages and may ultimately calcify. Calcification generally represents chronic, nonviable disease. In skeletal muscle, these calcified larvae appear as elongated densities aligned with the muscle fibers, giving rise to the classic “rice-grain” appearance on plain radiographs [4]. Because these calcifications are frequently an incidental finding, correctly recognizing the pattern is essential to avoid unwarranted investigation or treatment directed at an inactive process, particularly when a patient presents with symptoms that have another, more likely, explanation.

## Case presentation

An 85-year-old man was admitted with a viral respiratory infection complicated by respiratory failure and developed prominent diffuse lower-limb myalgia that was considered disproportionate to the clinical course of the infection. Physical examination was unremarkable, with no focal pain, focal muscle tenderness, palpable masses, or asymmetric limb edema, and there was no history of trauma. Deep vein thrombosis was considered given the immobilization associated with hospitalization and was excluded by lower-limb Doppler ultrasonography. Laboratory evaluation showed normal creatine kinase, inflammatory markers, renal function, and serum electrolytes, with no evidence of muscle injury or a metabolic cause for the myalgia. Given the prominence of the diffuse myalgia despite the absence of focal musculoskeletal findings, plain radiographs of the lower limbs were obtained to investigate a possible musculoskeletal cause.

The radiographs demonstrated numerous elongated soft-tissue calcifications, oriented parallel to the muscle fibers, distributed diffusely throughout the musculature of both lower limbs (Figures 1-4). This characteristic appearance was consistent with cysticercosis, a diagnosis that the patient's close relatives reported had previously been made. However, they were unable to provide details regarding the timing or method of the original diagnosis and did not know whether specific antiparasitic, corticosteroid, or surgical treatment had been administered; no documentation regarding previous diagnosis or treatment was available. Head computed tomography, performed for other reasons during the same admission, showed no evidence of neurocysticercosis. Because the calcifications were consistent with chronic, inactive disease, no further investigation of the musculoskeletal findings was pursued, and antiparasitic therapy was not considered, as it confers no benefit once the larvae have degenerated and calcified. The distribution and chronicity of the lesions, the normal laboratory evaluation, and the absence of focal musculoskeletal abnormalities did not support cysticercosis as the cause of the acute myalgia. The symptoms were instead attributed to a combination of the patient's advanced age, hospitalization-related immobilization, and the concurrent viral infection. The patient was managed conservatively with paracetamol for symptomatic relief, and the myalgia resolved completely within one week, in parallel with clinical recovery from the respiratory infection.

**Figure 1:**
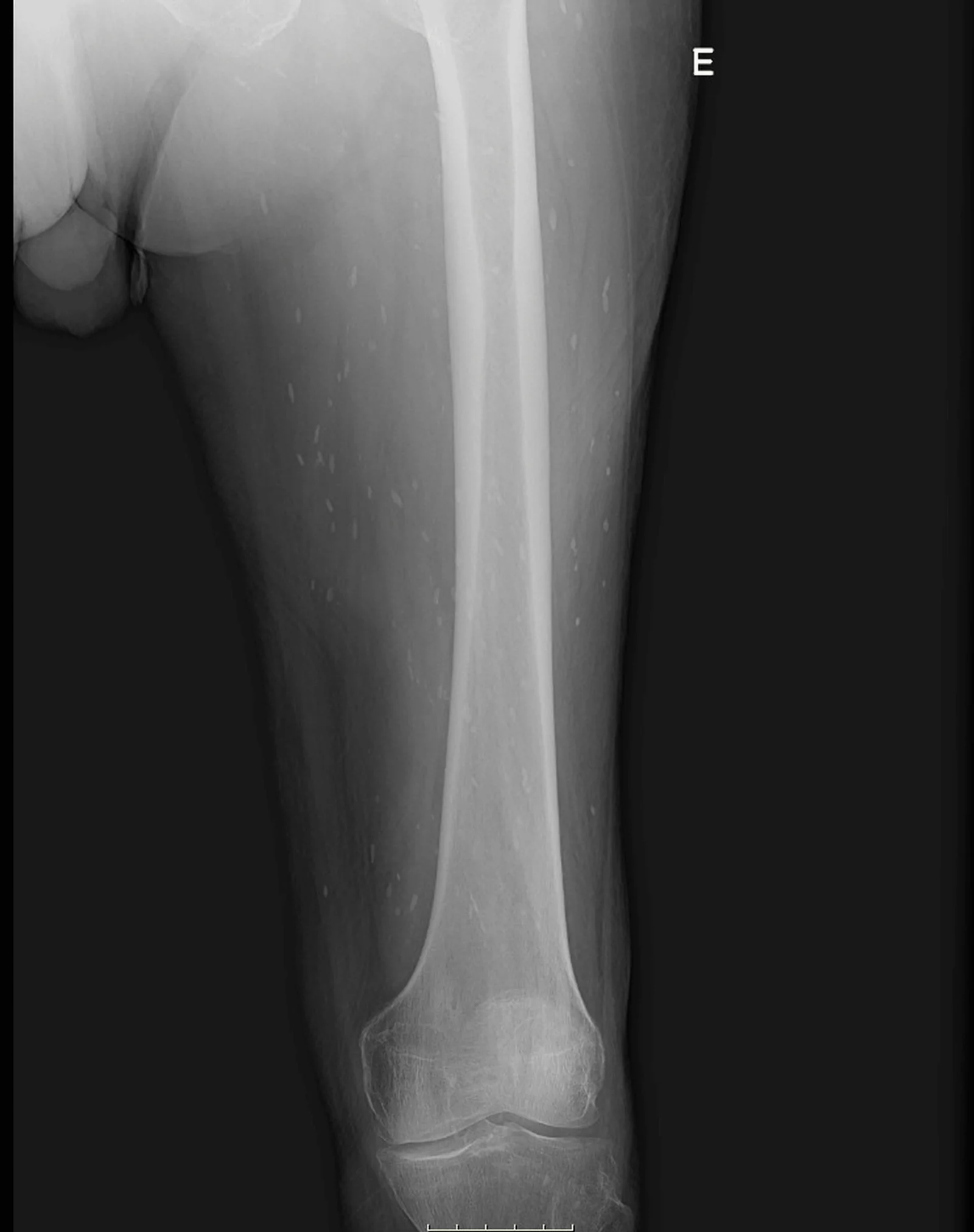
Anteroposterior radiograph of the left thigh and knee showing the same pattern of “rice-grain” calcifications.

**Figure 2:**
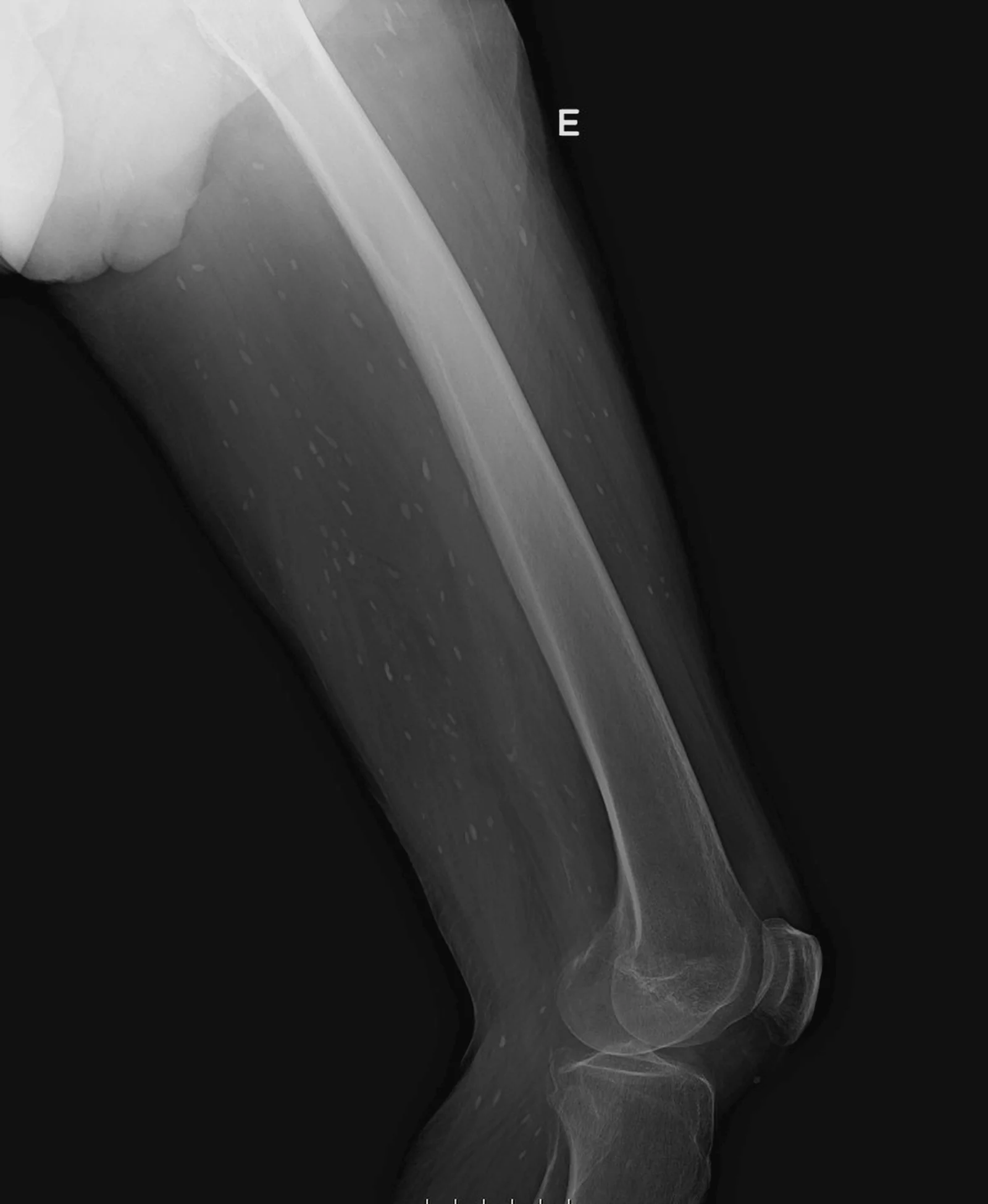
Lateral radiograph of the left thigh and knee, confirming the diffuse, muscle fiber-oriented distribution of the calcifications.

**Figure 3:**
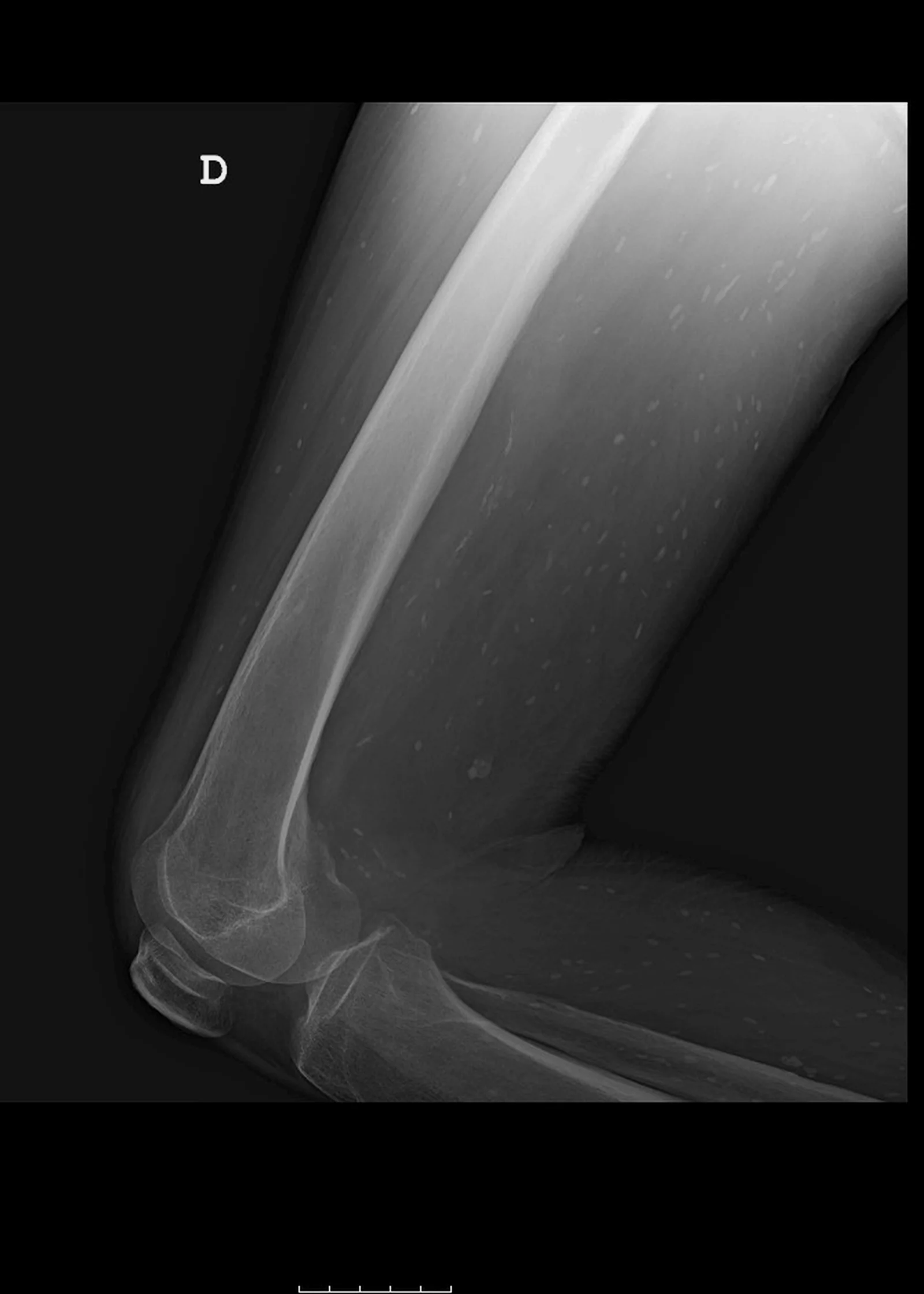
Lateral radiograph of the right knee, again demonstrating the characteristic elongated intramuscular calcifications.

**Figure 4:**
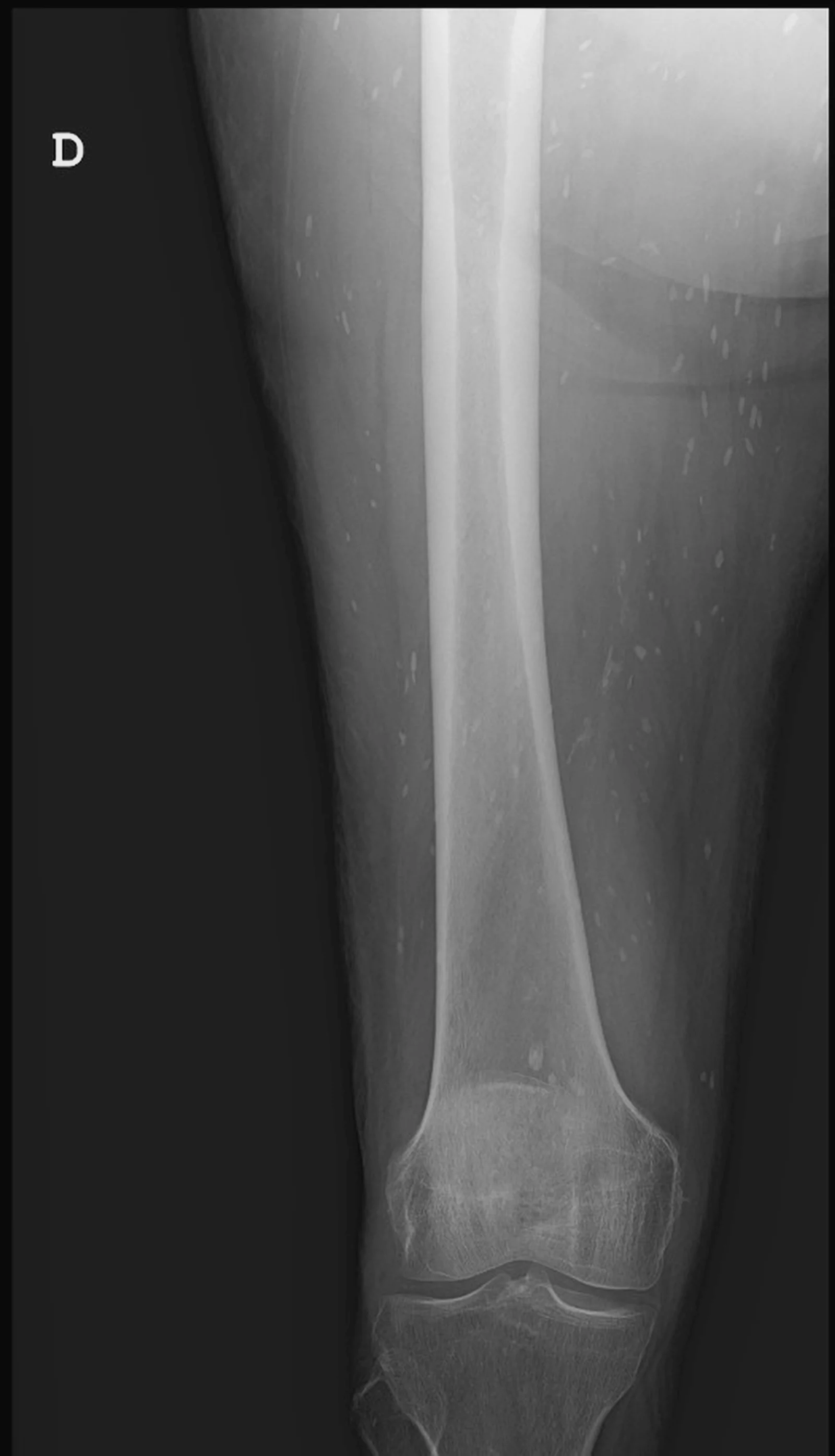
Anteroposterior radiograph of the right thigh and knee showing numerous elongated calcifications oriented parallel to the muscle fibers.

## Discussion

The diagnosis of muscular cysticercosis can frequently be established on the basis of its characteristic radiographic appearance alone, without the need for invasive investigation. Differential diagnoses for soft-tissue calcifications include myositis ossificans, calcinosis, tumoral calcinosis, and other dystrophic or metastatic calcifications, but the diffuse distribution of numerous small, elongated, muscle fiber-oriented densities is highly characteristic of cysticercosis and allows the two to be distinguished [4,5]. Antiparasitic treatment is generally reserved for viable cysts or symptomatic disease; in patients with calcified, inactive lesions such as this one, treatment is not recommended, as the larvae are no longer viable and therapy would not be expected to influence the clinical course [5].

This case also illustrates a broader diagnostic point: the presence of an incidental, unrelated radiographic abnormality can create a temptation to attribute a patient's acute symptoms to a chronic condition. Here, the extensive calcifications might have prompted unnecessary further work-up, or even inappropriate antiparasitic treatment, had they been mistaken for an active process. The absence of focal musculoskeletal abnormalities and the normal creatine kinase, inflammatory markers, renal function, and serum electrolytes provided no evidence of muscle injury, inflammation, or a metabolic cause. Recognizing both the chronic, inactive nature of the calcifications and their lack of correlation with the acute clinical picture allowed the myalgia to be attributed instead to a combination of more plausible factors: the patient's advanced age, the immobilization associated with hospitalization, and the underlying viral respiratory infection itself, a well-recognized cause of myalgia. Deep vein thrombosis, an important differential diagnosis in an immobilized hospitalized patient with limb symptoms, was considered and excluded by lower-limb Doppler ultrasonography. The complete resolution of symptoms within one week, in parallel with clinical recovery from the respiratory infection, supports this multifactorial attribution and indicates that the muscular calcifications played no role in the acute presentation.

## Conclusions

Recognition of the classic rice-grain calcification pattern allows prompt, noninvasive diagnosis of chronic muscular cysticercosis and helps prevent unnecessary investigations and inappropriate antiparasitic treatment in patients with inactive disease. Equally important, clinicians should be careful not to misattribute acute symptoms to such incidental chronic findings: in this patient, the clinical examination, normal laboratory evaluation, negative lower-limb Doppler ultrasonography, and clinical course supported the conclusion that the disproportionate myalgia was unrelated to the calcified cysticercosis and was instead explained by the combined effects of advanced age, immobilization, and the underlying viral respiratory infection, resolving with simple symptomatic treatment.
